# The Pneumonia Virus of Mice (PVM) Model of Acute Respiratory Infection

**DOI:** 10.3390/v4123494

**Published:** 2012-12-05

**Authors:** Kimberly D. Dyer, Katia E. Garcia-Crespo, Stephanie Glineur, Joseph B. Domachowske, Helene F. Rosenberg

**Affiliations:** 1 Laboratory of Allergic Diseases, National Institute of Allergy and Infectious Diseases, National Institutes of Health, Bethesda, MD 20892, USA; E-Mails: garciacrespoke@niaid.nih.gov (K.E.G.-C.); stephanie.glineur@nih.gov (S.G.); hrosenberg@niaid.nih.gov (H.F.R.); 2 Department of Pediatrics, SUNY Upstate Medical University, Syracuse, NY 13210, USA; E-Mail: domachoj@upstate.edu

**Keywords:** PVM, inflammation, leukocytes, eosinophils, respiratory syncytial virus, RSV, TLR, IFN, heterologous immunity, MIP-1α

## Abstract

Pneumonia Virus of Mice (PVM) is related to the human and bovine respiratory syncytial virus (RSV) pathogens, and has been used to study respiratory virus replication and the ensuing inflammatory response as a component of a natural host—pathogen relationship. As such, PVM infection in mice reproduces many of the clinical and pathologic features of the more severe forms of RSV infection in human infants. Here we review some of the most recent findings on the basic biology of PVM infection and its use as a model of disease, most notably for explorations of virus infection and allergic airways disease, for vaccine evaluation, and for the development of immunomodulatory strategies for acute respiratory virus infection.

## 1. Introduction

Pneumonia virus of mice (PVM), human respiratory syncytial virus (hRSV) and bovine respiratory syncytial virus (bRSV) are enveloped, negative sense, single-stranded RNA viruses of the family *Paramyxoviridae*, subfamily Pneumovirinae, genus Pneumovirus [1]. PVM was originally discovered in 1939 by researchers Horsfall and Hahn at The Rockefeller University as part of an attempt to identify pathogens from human clinical samples that would replicate in lung tissues of inbred mice. PVM was isolated from lung tissue of what had been presumed to be healthy control mice that had been subjected to serial mouse-to-mouse passage [2]. PVM virions are polymorphic and found in diverse shapes, from spheres of 80–120 μm in diameter to filaments up to 3 μm in length. The virus replicates over a period of 24–30 hours in mouse lung tissue, with virus amplification proceeding at 16-fold per cycle [2].

PVM is one of the many virus pathogens that are monitored in commercial and research rodent colonies [3,4]. In a study covering the years 2004–2007, Liang and colleagues [5] reported that 0.2%–1.0% of isolates from mouse colonies and 6.4%–25.8% of isolates from rat colonies tested were positive for PVM. Information on wild rodents is somewhat limited. However, an extensive three-year study performed by Kaplan and colleagues [6] documented over 40% seropositivity for PVM in nearly 300 small wild rodents tested at 11 field sites in the United Kingdom. In contrast, Smith and colleagues [7] found no seropositivity for PVM among wild house mice in Southern Australia. 

**Figure 1:**
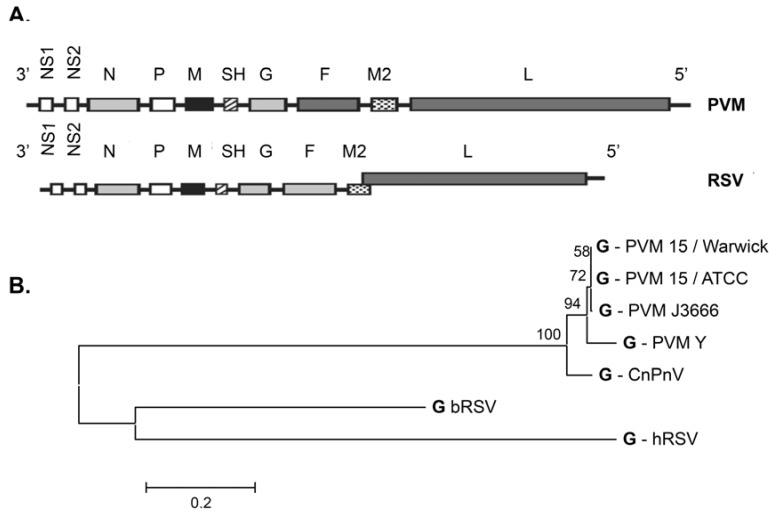
(**A**) Although there is little direct amino acid sequence homology between PVM and hRSV, the two viruses share the same gene order. (**B**) Neighbor-joining tree featuring the amino acid sequences of the G glycoproteins of selected pneumoviruses; Genbank accession numbers include FJ614813.1; NC_001989.1; NC_006579; AY729016.1; JQ899033.1; HQ734815; AY743910.1. Panel A reprinted with permission from [1].

It is not yet clear how or if PVM replicates and induces pathology in non-rodent hosts. In a study carried out in 1986, Pringle and Eglin [8] found that more than 75% of adult sera had PVM‑neutralizing activity that did not correlate with hRSV or parainfluenza virus (PIV)-3 neutralizing activity. More recently, Brock and colleagues [9] explored this question further, and determined that PVM did not replicate *in situ* when administered to the respiratory tracts of non-human primates, and that the PVM-neutralizing factor(s) in human sera did not interact specifically with virion components. In another recent development, Dubovi and colleagues [10,11] reported the isolation of canine pneumovirus (CnPnV) from the respiratory tracts of shelter-confined dogs with apparent respiratory illness. CnPnV is very similar overall to PVM (Figure 1), replicates in the lungs of BALB/c mice and induces inflammatory pathology, morbidity and mortality similar to that elicited by PVM [12] but a much higher initial inoculum is required to elicit these effects. The specific virulence attributable to this virus in canine species remains to be explored. 

There are two characterized strains of PVM, strain 15 (two variants) and strain J3666 in current use in the research community. The original studies by Horsfall and co-workers [2,13,14,15] were performed on an isolate named strain 15, which was reported to be highly pathogenic in mice. Since that time, this strain had reportedly undergone tissue-culture passage, resulting in loss of its pathogenicity *in vivo*. Strain 15/Warwick is highly attenuated and elicits minimal inflammatory response [16] while strain 15/ATCC (American Tissue Culture Collection VR-25), in our hands, elicits inflammatory pathology in BALB/c mice but substantially less disease pathology in C57BL/6 mice [17]. Strain J3666 has reportedly been maintained via mouse passage [18] and thus retains full pathogenicity. The molecular organization of the PVM genome was elucidated by Easton and colleagues [19,20,21,22] and Krempl and colleagues [23,24]. The most significant differences between strains 15 and J3666 are in the G attachment protein. Anh and colleagues [25] documented the susceptibility of various strains of mice to strain J3666 as follows: 129/Sv > DBA > C3H/HeJ > BALB/c > C57BL/6 > SJL. Glineur and colleagues [26] have recently explored PVM infection in crosses between 129/Sv and SJL mice and have documented the polygenic nature of resistance and susceptibility to severe virus infection. A third strain, PVM strain Y, originally derived from a spontaneous infection in athymic mice [27] and featured in an early study of disease exacerbation in mice with severe combined immunodeficiency disease [28] has recently been sequenced (Figure 1; [29]). 

Horsfall and Ginsberg [14] recognized the potential of PVM for the exploration of acute respiratory virus infection in an evolutionarily relevant host. These authors were also the first to relate the development of lung lesions to ongoing virus replication and to evaluate altered morbidity and mortality in response to immunomodulatory therapy, specifically in response to administration of bacterial capsular polysaccharide [13,14,15]. We, and others, are using the PVM infection model to study the importance of virus-induced inflammatory responses in the development of severe respiratory virus disease and as a platform for the development of novel immunomodulatory strategies (see section on PVM and Disease, Heterologous Immunity).

## 2. The PVM Model of Acute Respiratory Infection

Our initial studies on PVM focused on the inflammatory responses to respiratory virus infection in a natural, evolutionarily relevant host [30,31]. We reproduced the findings of Horsfall and colleagues and reported robust virus replication *in situ* (to titers >10^8^ pfu/gm lung tissue), progressing to marked morbidity (hunching, fur ruffling), weight loss, and mortality in response to a minimal virus inoculum of the highly pathogenic strain PVM J3666 [32,33]. We have localized immunoreactive PVM to the bronchiolar epithelium [34], in a distribution similar to what has been observed for RSV in human post-mortem specimens [35]. Profound inflammation of the lungs is evident and especially noteworthy is the recruitment of granulocytes and severe pulmonary edema. PVM replication in the mouse lung tissue is associated with local production of proinflammatory mediators including MIP-1α, MIP-2, and MCP-1 [34], consistent with those detected in lung and nasal washings in association with the more severe forms of RSV disease in human infants [1,36]. Although some features of the PVM model clearly conform to human pathophysiology, others do not. For example, neonatal mice exhibit little to no overt inflammation in response to PVM infection [37], nor can we establish a distinct pattern of infection in aged mice [38]. Similarly, it is crucial to recognize that PVM has no direct cross‑reactivity with the human RSV pathogen, thus one’s ability to perform studies of antigen-specific acquired immunity are limited. 

## 3. Host Immune Response to PVM Infection

### 3.1. Neutrophils and Eosinophils

**Figure 2:**
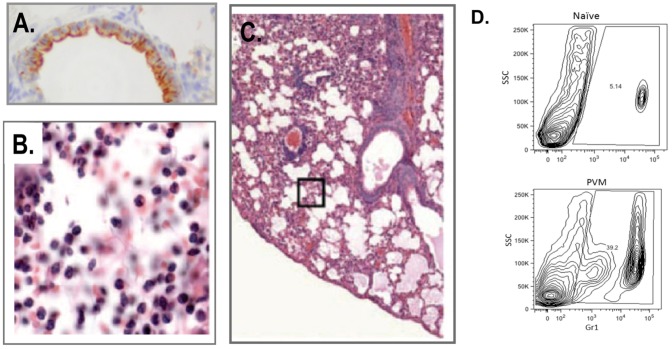
(**A**) Detection of PVM in bronchiolar epithelial cells, original magnification 63×; (**B**,**C**) Histology of lung tissue from PVM-infected wild-type C57BL/6 mice, featuring multifocal acute alveolitis with intra-alveolar edema with scattered hemorrhage and moderate granulocytic infiltrates throughout; original magnifications 63× and 20×, respectively; (**D**) Flow cytometric profiles of Gr1^+^ granulocytes in single cell suspensions from lung tissue of naïve and PVM-infected BALB/c mice. Reprinted with permission from (A) [34]; (B) and (C) [54].

Microscopic examination of bronchoalveolar lavage fluid and lung tissue from morbid mice reveals profound inflammation, most notable for recruitment of granulocytes and progression to pulmonary edema (Figure 2). Similar to findings from the mouse model of influenza virus [39], MIP-1α signaling through CC chemokine receptor (CCR)-1, its major receptor on neutrophils and eosinophils, is crucial for granulocyte recruitment in response to PVM infection [33]. We have built on this observation to explore immunomodulatory therapies for pneumovirus infection directed at limiting uncontrolled neutrophil influx [40,41] as discussed below. 

The role of eosinophils in respiratory virus infection is controversial and somewhat of a “double-edged sword” (reviewed in [42,43,44]). Eosinophils are among the granulocytes recruited at the earliest time points in response to PVM infection [32]. We and others have shown that eosinophils have antiviral properties against RSV [32,45,46]; recent findings from our laboratory demonstrate that activated eosinophils promote survival against lethal PVM infection [47]. PVM replicates in mouse eosinophils and promotes cytokine release [48].

### 3.2. T Lymphocytes

Although T cells have no apparent impact on the outcome of acute lethal PVM infection, both CD4^+^ and CD8^+^ T cells are required for virus clearance in response to sublethal infection [49]. Claassen and colleagues [50] documented influx of activated CD8^+^ T cells into the lungs of infected mice and characterized PVM-specific responses against epitopes in the virus M (matrix; M_43–51_), F (fusion; F_304–312_) and P (phosphoprotein; P_261–269_) virion proteins. The relatively limited frequency of functional virus-specific CD8^+^ T cells suggested that PVM infection resulted in inactivation of effector T cells, similar to what has been reported in acute RSV infections [51]. Claassen and colleagues [52] have also identified a CD4^+^ T cell epitope in the G attachment protein, G_381–385_ and demonstrated protective immunity against lethal PVM challenge when mice were immunized simultaneously with both the CD4^+^ G_381–385_ and the P_261-269_ CD8^+^ T cell epitope peptides. 

While CD4^+^ and CD8^+^ T cells have been reported to promote virus clearance, IL-21, a type I cytokine produced primarily by activated CD4^+^T cells, promotes pathology in response to PVM infection [53]. Mice devoid of the unique receptor for IL-21 (IL21R^−/−^) have diminished levels of the proinflammatory chemokine KC, and recruit fewer neutrophils, CD4^+^, CD8^+^ and gamma-delta T cells to the lungs, and survive longer in response to PVM infection than their PVM-infected wild-type counterparts.

### 3.3. Macrophages

Macrophages are the main resident phagocytes in the lung and, working together with intact muco‑ciliary to clear unwelcome debris including pathogens. Rigaux and colleagues [55] showed that depletion of alveolar macrophage prior to PVM infection resulted in a small increase in virus recovery and paradoxically prolonged survival. Macrophage depleted PVM-infected mice exhibited enhanced NK cell recruitment to the lungs accompanied by increased production of IFNγ by recruited NK cells, CD4^+ ^and CD8^+^ T cells. Interestingly, in similar studies featuring RSV challenge, Pribul and colleagues [56] found that macrophage depletion had no impact on virus-mediated T cell recruitment, weight loss or lung function, while Reed and colleagues [57] found that macrophage depletion prior to RSV challenge resulted in prominent airway occlusion in association with ongoing disease.

### 3.4. Toll-Like Receptors

Several studies have elucidated the nature and function of pattern recognition receptors (PRRs) that are involved in initiating the immune response to hRSV [58,59,60]. Despite findings that focused on hRSV signaling via TLR4, Faisca and colleagues [61] found that the sequelae of PVM infection—specifically, virus recovery, histopathology, body weight, and pulmonary function—were indistinguishable from one another when examined in wild-type and TLR4 gene-deleted mice. The results with the PVM model are consistent with recent findings from Marr and Turvey [62] who found that NF-kB activation mediated by infectious RSV particles in cell culture does not require the presence of a functional human TLR4/MD-2/CD14 complex.

Davidson and colleagues [63] utilized the PVM model to explore the role of TLR7 in promoting host defense against acute pneumovirus infection. Among their findings, PVM infection in TLR7 gene-deleted mice was associated with delayed induction of interferons and diminished recruitment of NK cells and neutrophils; adoptive transfer of TLR7-sufficient plasmacytoid dendritic cells restored innate antiviral responses and promoted virus clearance. Interestingly, TLR7-sufficient eosinophils also promote virus clearance in mouse models of RSV challenge [46].

### 3.5. Type I Interferons

Pneumoviruses have developed an efficient strategy to circumvent the host IFN response (reviewed in [64]). Among the most prominent of these findings, the non-structural NS1 and NS2 proteins of both human and bovine RSV inhibit the IFN alpha and beta (type I IFN) signaling pathways via several independent mechanisms [65,66,67,68], including degradation of the STAT2 signaling intermediate and blockade of activation and nuclear translocation of the transcription factor, interferon-regulatory factor 3. 

Garvey and colleagues [54] were the first to evaluate the interactions of PVM and type I interferons in their study of the sequelae of virus infection in mice devoid of the receptor for type I interferons (IFNαβR^−/−^ mice). PVM infection clearly elicited preferential expression of a wide spectrum of interferon-regulated and interferon-response genes, and virus replication *in vivo* was relatively suppressed in wild-type *vs.* IFNαβR^−/−^ mice. However, paradoxically prolonged survival was observed among the IFNαβR^−/−^ mice, which may be attributed to the overriding impact of differential inflammatory pathology. Among the most striking differences, the IFNαβR^−/−^ mice developed tertiary mucosal associated lymphoid tissue (MALT, or B (bronchus) ALT) which has been associated with protection against virus pathogens in other settings (reviewed in [69]).

There are two studies that have directly addressed the role of PVM NS1 and NS2 using the recombinant virus (rPVM). In the first, Buchholz and colleagues [70] identified both NS1 and NS2 as virulence factors, as rPVMΔNS1 (*i.e.*, with the NS1 gene-deletion), and more notably, rPVMΔNS2, and rPVMΔNS1ΔNS2 replicate less efficiently in BALB/c mice than the parent wild-type rPVM, and resulted in fewer clinical symptoms. Interestingly, the ΔNS1 elicited production of both IFNα and IFNβ was indistinguishable from that of the parent rPVM; ΔNS2 and ΔNS1ΔNS2 gene-deleted viruses elicited higher levels of both IFNα and IFNβ than the parent rPVM at early time points during infection. In the second study, Heinze and colleagues [71] found that all three of the aforementioned deletion mutants replicated more effectively in IFNαβR^−/−^ mice than they did in wild type mice; replication of the mutant viruses was further enhanced in mice devoid of both IFNαβR and IL28Rα, the receptor for the type III interferon, IFNλ. 

## 4. Using the PVM Model to Explore Human Disease

### 4.1. Inflammation and Acute Infection

Among the primary reasons to explore respiratory virus infection using the PVM model is to improve our understanding of the molecular basis of severe disease so as to design novel therapeutic strategies. As a natural rodent pathogen, PVM undergoes robust virus replication in lung tissue [31]. However, we have found that even highly effective antiviral therapy—strategies such as systemic ribavirin that result in immediate cessation of all further virus replication—do not provide tangible benefits when one is evaluating morbidity and mortality as endpoints (reviewed in [72]). Indeed, our experience with the PVM model mirrors the human clinical observations with ribavirin use for RSV infection. Ribavirin was once administered routinely to infants hospitalized with RSV disease; and although it was quite effective as an antiviral, clinical benefits were not observed [73,74]. As such, the PVM model has provided the impetus to explore several specific immunomodulatory strategies. Among the most promising directions are combined antivirals and immunomodulatory blockade of the proinflammatory cytokine, MIP-1α [40,41]. Specifically, we have shown that antibody-mediated blockade of the actions of MIP-1α resulted in improved survival, from 20% in response to ribavirin alone to 60% in response to ribavirin together with anti-MIP-1α monoclonal antibody. Survival in response to acute PVM infection was also enhanced in response to increasing concentrations of metRANTES, which blocks MIP-1α signaling via its primary receptor, CCR1. 

A similar study documented the effectiveness of ribavirin in PVM infection in conjunction with montelukast a cysteinyl-leukotriene inhibitor [75]. Neither agent was effective at reducing morbidity or mortality when administered alone. However, significant improvements in long-term survival were observed when provided as combined therapy. Interestingly, montelukast had little impact on neutrophil recruitment, suggesting that the presence of neutrophils alone does not indicate inevitable progression to intractable disease.

The chemerin/ChemR23 (also known as CMKLR1) pathway is another potential therapeutic target [76]. pDCs preferentially express chemerin and prochemerin is processed by neutrophil proteases. Given that pDCs and neutrophils play an important role in the physiopathology of viral infections of the lung, the role of chemerin/ChemR23 in PVM was investigated. PVM-infected ChemR23^−/−^ mice responded with augmented neutrophil recruitment, delayed virus clearance, and higher rates of morbidity and mortality than wild type counterparts, a response suggesting the therapeutic value of supplementation with the activated adipokine, chemerin, during acute virus infection.

Glucocorticoids are in general use as potent and non-specific anti-inflammatory agents but have only limited benefit for the treatment of severe hRSV-associated inflammation [77,78,79,80]. We have shown that hydrocortisone therapy has no effect on the production of MIP-1α or on the influx of neutrophils during acute severe PVM infection. In fact, PVM-infected mice responded to hydrocortisone with enhanced viral replication and slightly accelerated mortality [81] suggesting that the added immunosupression of glucocortcoids in this context contributed to illness severity.

Bem and colleagues [82] explored the impact of mechanical ventilation on the acute inflammatory response in mice infected with PVM. In addition to increased levels of cytokines in the airways, mechanical ventilation activated caspase-dependent cell death pathways leading to acute lung injury in PVM-infected mice. In a subsequent study, van den Berg and colleagues [83] found that inflammatory injuries associated with mechanical ventilation were less severe in *lpr* (Fas-deficient) mice although all mice ultimately succumbed to infection.

### 4.2. Asthma and Allergic Airway Disease

The role of respiratory virus infection in promoting and exacerbating asthma and existing respiratory allergies is an area of significant medical concern [84,85]. Siegle and colleagues [86,87] have used PVM to determine how recovery from a respiratory virus infection early in life might alter subsequent responses to an unrelated allergen. Mice that recovered from a sublethal PVM infection displayed an exaggerated Th2 response to a chronic intranasal ovalbumin sensitization followed by a moderate challenge, with elevated levels of serum IgE and augmented expression of IL-4, IL-5 and IL‑13; these responses were suppressed by a combination of neutralizing antibodies against both IL-4 and IL-25. Similarly, Barends and colleagues [88] found that PVM could exacerbate an ongoing allergic response. When PVM was administered to sensitized mice together with an intranasal antigen challenge, the virions elicited augmented eosinophil recruitment together with local elevation of Th2 cytokines. 

### 4.3. Vaccines

The first RSV vaccination trial, performed in the early 1960s with a formalin-inactivated preparation (FI-RSV, lot 100) resulted in an aberrant deleterious response following natural hRSV infection. Numerous vaccinated infants developed severe respiratory complications from subsequent natural RSV infection including two deaths, a phenomenon later referred to as “enhanced disease” [89]. Enhanced disease has been studied extensively, and has been modeled in BALB/c mice inoculated with formalin-inactivated RSV and RSV virion components (reviewed in [90,91]). PVM antigens, when prepared and administered in a manner analogous to the hRSV lot 100 vaccine also induces the enhanced disease response, likewise characterized by elevated levels of Th2 cytokines and eosinophil recruitment to airways and lung tissue [92]. Interestingly, the eosinophils, long perceived to be the cells promoting respiratory pathology in this setting, had no impact on virus recovery or weight loss in this experimental model. 

Enhanced disease observed during the FI-RSV lot 100 study was among the issues that constrained further progress in the development of an RSV vaccine. Now, several decades later, a small number of human infant RSV vaccine trials are underway. Among the current vaccines under study, recent success with recombinant rodent Sendai virus (SeV) used to deliver RSV antigens [93,94] suggests that a similar approach may be feasible utilizing recombinant PVM [9,71]. Most recently, van Helden and colleagues [95] used the PVM model to explore the role of antigen-specific CD8^+^ T cells as a useful vaccine strategy. Among their findings, adoptive transfer of PVM-specific CD8^+^ T cells do provide at least partial protection against acute pneumovirus disease, and do not appear to contribute to immunopathology. 

### 4.4. Heterologous Immunity

**Figure 3:**
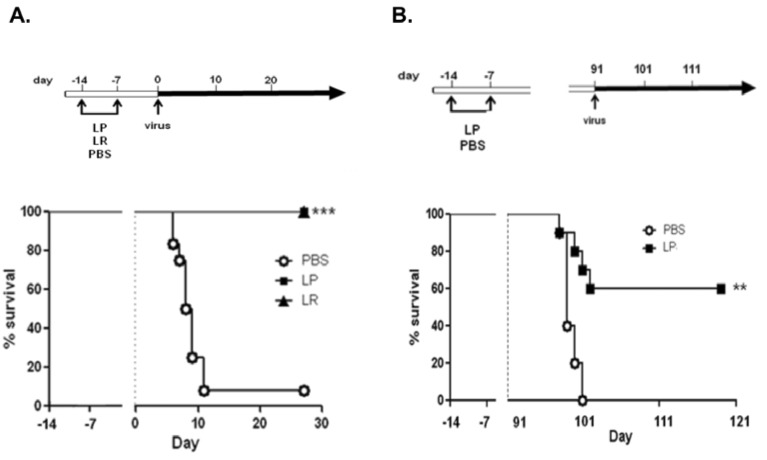
(**A**) Mice primed via intranasal inoculation with *L. plantarum *or *L. reuteri* are fully (100%) protected from the lethal sequelae of PVM infection. (**B**) Prolonged survival and significant long-term protection results even when virus challenge was delayed until 91 days (3 months) after initial *Lactobacillus*-mediated priming. Reprinted with permission from [96].

As part of an exploration of the immunomodulatory potential of probiotic *Lactobacillus* strains, we found that wild-type mice primed via intranasal inoculation with Lactobacillus plantarum or Lactobacillus reuteri were fully protected against lethal sequelae of a subsequent PVM infection ([96], Figure 3). These findings are a particularly robust example of heterologous immunity, a concept recently introduced into the literature that explains observations such as this, in which increased resistance (or susceptibility) to an unrelated (*i.e.*, not cross-reactive) pathogen can be observed upon recovery from an inflammatory insult [97,98,99]. There are a number of examples in which PVM has been featured as a target pathogen in studies of heterologous immunity. One such study is that of Wiley and colleagues [100] who elicited protection against PVM (within a larger series of respiratory viruses) via instillation of protein cage nanoparticles, which are multi-subunit homopolymers of unique heat shock proteins from the thermophilic bacterium, *Methanocaldococcus jannaschii*.) Likewise, Easton and colleagues [101] found that inoculation of mice with the defective interfering (DI) deletion mutant influenza 244/PR8 protects against subsequent infection with PVM. Interestingly, although each of these initial priming events—*Lactobacillus*, nanoparticles or defective interfering virus—all lead to a shared outcome, specifically, protection from the lethal sequelae of PVM infection, the cellular and biochemical mechanisms promoting these responses are unique and stimulus-specific [102].

## 5. Conclusions

The PVM model holds great promise for the elucidation of inflammatory mechanisms associated with pneumovirus infection. Studies carried out to date have provided a rationale for the use of chemokine and/or chemokine receptor blockade alone and/or in conjunction with appropriate antiviral therapy as a means to reduce the inflammatory pathology in severe pneumovirus disease. Likewise, PVM is an excellent system in which to explore the molecular mechanisms of heterologous immunity to pneumovirus infection, information that may assist in the development of vaccines and other novel prevention strategies.
